# MGAT1 is a novel transcriptional target of Wnt/β-catenin signaling pathway

**DOI:** 10.1186/s12885-017-3960-7

**Published:** 2018-01-08

**Authors:** Izzet Akiva, Necla Birgül Iyison

**Affiliations:** Department of Molecular Biology and Genetics, Institute of Graduate Studies in Science and Engineering, Boğaziçi University, İstanbul, Turkey

**Keywords:** Hepatocellular carcinoma, Wnt/β-catenin pathway, MGAT1, Xenografting, SCID mice

## Abstract

**Background:**

The Wnt/β-catenin signaling pathway is an evolutionary conserved pathway, which has important functions in vertebrate early development, axis formation, cellular proliferation and morphogenesis. Additionally, Wnt/β-catenin signaling pathway is one of the most important intracellular pathways that controls cancer progression. To date most of the identified targets of this pathway are shown to harbor tumorigenic properties. We previously showed that Mannosyl glycoprotein acetylglucosaminyl-transferase (MGAT1) enzyme is among the Wnt/β-catenin signaling putative target genes in hepatocellular carcinoma cell lines (Huh7).

**Methods:**

MGAT1 protein levels were determined by Western Blotting from Huh7 cell lines in which Wnt/β-catenin pathway was activated by means of different approaches such as LiCl treatment and mutant β-catenin overexpression. Luciferase reporter assay was used to analyze the promoter activity of MGAT1. The mRNA levels of MGAT1 were determined by quantitative real-time PCR from Huh7 cells that were treated with either Wnt agonist or GSK-3β inhibitor. Wound healing and XTT cell proliferation assays were performed in order to determine the proliferation and migration capacities of MGAT1 overexpressing stable Huh7 cells. Finally, xenograft experiments were carried out to measure the tumor formation capacities in vivo.

**Results:**

In this study we showed that the activation of Wnt/β-catenin pathway culminates in the upregulation of MGAT1 enzyme both at transcriptional and post-transcriptional levels. We also showed that overexpression of the β-catenin gene (CTNNB1) increased the promoter activity of MGAT1. We applied a set of complementary approaches to elucidate the functional importance of MGAT1 as a vital target of Wnt/β-catenin signaling in Huh7 cells. Our analyses related to cell proliferation and migration assays showed that in comparison to the control cells, MGAT1 expressing Huh7 cells have greater proliferative and invasive capabilities. Furthermore, the stable overexpression of MGAT1 gene in Huh7 cell lines lead to a significant increase in tumor growth rate in Severe Combined Immunodeficient (SCID) mice.

**Conclusions:**

Taken together, we showed for the first time that MGAT is a novel Wnt/β-catenin pathway target that has important implications for tumorigenesis.

## Background

The Wnt Signaling pathway is conserved in various organisms from worms to mammals, and play important roles in development, differentiation, cellular proliferation, morphology, motility and fate [1]. Wnt proteins constitute a family of secreted cysteine-rich glycoproteins that exhibit distinct expression patterns in embryo and adult organisms [2, 3]. Mechanistically, binding of the Wnt protein to its receptors stimulates 3 different pathways; which are canonical Wnt pathway, planar cell polarity pathway (PCP) and Wnt/calcium pathway [4].

The canonical Wnt/β-catenin signaling pathway is the best understood Wnt signaling pathway. β-catenin, which is associated with the membrane bound E-cadherin, or is free in the cytoplasm, plays a major role in the transduction of the canonical Wnt/β-catenin signal [5, 6]. Cytoplasmic β-catenin levels are normally kept low through continuous proteasome-mediated degradation, which is controlled by a multiprotein complex containing Glycogen synthase kinase 3/Adenomatous polyposis coli/Axin (GSK-3β/APC/Axin). The canonical Wnt/β-catenin signaling pathway is initiated by the binding of a Wnt ligand to the Frizzled receptor. Here it progresses through sequential events leading to the stabilization and translocation of β-catenin into the nucleus where it interacts with the TCF/LEF family of transcription factors in order to activate target gene expression [7]. Several targets of the Wnt/β-catenin pathway were identified as genes that regulate cell proliferation, development and are involved in tumor progression [8].

In various types of cancers (colon, liver, prostate, blood, skin) Wnt/β-catenin associated molecules, such as proto-oncogene CTNNB1, and tumor suppressor genes such as APC or AXIN show alterations [9]. Among these, hepatocellular carcinoma and colorectal carcinoma harbor the highest rate of Wnt pathway gene mutations. In addition to these mutations, the nuclear accumulation of β-catenin due to aberrant activation of Wnt signaling is mainly observed in hepatocellular, colorectal, gastrointestinal, and breast tumors [10].

In a quest for Wnt signaling targets we identified MGAT1 (Mannosyl (alpha-1,3-)-glycoprotein beta-1,2-N-acetylglucosaminyl-transferase), which is a medial Golgi enzyme that catalyzes the first step in the conversion of oligomannose-type N-glycans into complex and hybrid N-glycans [11]. The deduced protein sequence of MGAT1 is 445 amino acids in length, and it has general characteristics of other Golgi transferases that are type II transmembrane proteins [12].

MGAT1 is essential for normal embryogenesis in the mouse. Previous studies showed that mice lacking a functional MGAT1 gene die at approximately 10 days after fertilization with multiple developmental abnormalities, indicating the importance of complex and hybrid type N-glycans in cell-cell interactions [13].

Null mutations in Drosophila MGAT1 have been shown to produce defects in adult locomotory activity when compared to wild type flies. Moreover, the null mutant males are sterile and determined to have a severely reduced mean life span. Thus it is stated that, MGAT1-dependent N-Glycans are required for locomotory activity, life span and brain development in flies [14].

In our previous study [15], we performed SAGE (Serial Analysis of Gene Expression) and genome-wide microarray techniques in order to screen for novel Wnt/β-catenin pathway targets by overexpressing a degradation-resistant β-catenin mutant (S33Y-β-catenin) in Huh7 (hepatocellular carcinoma) cell lines, which lack detectable levels of nuclear endogenous β-catenin. In these screens, we found MGAT1 gene to be differentially expressed and, in this study we show that MGAT1 is a transcriptional target of the Wnt/β-catenin pathway. Our studies indicate that MGAT1 is upregulated at both RNA and protein levels in response to Wnt/β-catenin pathway activation. Further in vitro and in vivo experiments also show that overexpression of MGAT1 can play a role in tumor progression.

## Methods

### Cell culture

Cell lines were cultured in Dulbecco’s modified Eagle’s medium (Hyclone) containing 10% fetal bovine serum (Gibco) and 1% penicillin/streptomycin (Hyclone) at 37 °C in 5% CO_2_. Passages 6–15 were used for the experiments. Cells were routinely passaged before reaching ~90% confluence. Transfections were done using the Turbofect transfection reagent (Thermo) according to DNA: reagent ratios suggested by the manufacturer.

Huh7 cells (Cat no. PTA-4583) were treated with Wnt agonist (Calbiochem) and GSK-3β Inhibitor XII, TWS 119 (Calbiochem) in concentration-dependent manner. DMSO was used as negative control.

For the generation of stable cell lines; Huh7 cells in 6-well plates were transfected separately with the following constructs: pIRES2-EGFP-MGAT1 and pIRES2-EGFP-GFP (control). Transfected cells were cultivated in the presence of G-418 geneticin sulfate solution (Hyclone). 500 μg/ml geneticin was used as working solution for selection of stable cell lines. The selection was carried on for 3–4 weeks, while the cells were passaged when confluency was reached. 250 μg/ml geneticin was used as working solution for the maintenance of stable cell lines.

### Wound Healing Assay

Huh7 cells stably expressing the corresponding genes were grown to full confluency on a 6-well cell culture plate. A wound is introduced by scratching the confluent monolayer cells with a pipette tip (time = 0). Plates were washed twice with 1X PBS in order to remove detached cells and incubated with complete growth medium. Cell migration into the scraped empty space was followed at 24 h, 48 h and 72 h time intervals using bright field microscopy. At the end of 72 h, the distances across the wound were quantified by using the ImageJ software.

### Xenograft experiments

Stable Huh7 cells were cultivated in DMEM without any antibiotics for 48 h before in vivo studies. For tumor formation assay, the confluent cells in 10-cm culture plates were trypsinized and washed twice with PBS. The cell pellets were resuspended in 150 μl PBS and kept on ice until injection. The cells were injected subcutaneously and bilaterally (control on one side) into the abdominal region of 6–14-week-old female SCID mice. Tumors at the injection sites were collected at different days post-injection to determine tumor volume and weight. The isolated tumors were snap-frozen in liquid nitrogen and stored at −80 °C for further use.

### Total RNA extraction

Total RNA isolation was performed with GeneJet RNA Purification kit (Thermo) following the manufacturer’s instruction. Adherent cells were washed with PBS, trypsinized and centrifuged at 1600 rpm for 5 min. Cell pellets were dissolved in 600 μl lysis buffer and vortexed for 10 s. Homogenization of the lysate was accomplished by passing through a RNase-free syringe several times. Total RNA concentration was measured by nanodrop and absence of DNA contamination is verified (OD 260/280 ratio > 2). Total RNA integrity was checked by loading the samples on 1% agarose gel and confirming the presence of 18S and 28S ribosomal RNAs. Purified RNA samples were stored at −80 °C until use.

### Luciferase Reporter Assay

Luciferase assay was performed according to Dual-Glo Luciferase Assay System (Promega) protocol. For the assay, cells were co-transfected with 1 μg of a pGL3-luciferase reporter plasmid including promoter of interest, 1 μg β-catenin, TCF4 or dNTCF4 expression plasmid and 100 ng of pGL3-SV40-Renilla (internal control) per well of a 12-well plate. Transfection is done by using Turbofect transfection reagent (Thermo) according to the manufacturer’s instructions. About 48 h post-transfection, cells were collected by trypsin, and then washed with 1X PBS twice. Pellet was completely resuspended in 100 μl 1X PBS and placed into 96-well plate. Firefly and Renilla luciferase activities were measured respectively, using a fluorometer. Firefly luciferase readings were normalized to Renilla luciferase readings and graphs were plotted in Microsoft Excel.

### Lithium Treatment Assay

Huh7 cells were seeded into 10 cm culture dishes or 6-well plates based on the experiments. Following day, lithium chloride (LiCl) or sodium chloride (NaCl) was added to the medium of the cells to a final concentration of 25 mM. Reverse treatment was employed where all the cells treated at different time points were harvested at the same time (*t* = 0 h). For protein isolation from the treated cell lines, cell lysis buffer with freshly added protease inhibitors was added on to the cells (100 μl for per well of 6-well plate and 400 μl for 10 cm plate) and the cells were subjected to protein extraction procedure.

### Quantitative Polymerase Chain Reaction

Real-Time PCR was performed with gene-specific primers using the Maxima SYBR Green/ROX qPCR kit (Thermo) according to the manufacturer’s protocol. The primers were chosen among the primers of MGH primer bank encompassing intron/exon junctions and blasted against the target genome. The reaction mixtures were distributed into the wells of 96-well qPCR plate and cDNAs were added last. The plate was centrifuged at 2000 rpm for 2 min in order to collect the components to the bottom of the wells. PCR reaction was started with initial denaturation at 95 °C for 10 min. Amplification cycle consists of three steps; a denaturation step at 95 °C for 15 s, an annealing step at 60 °C for 30 s and an elongation step at 72 °C for 30 s. It was repeated for 40 cycles and finished by a melting curve step. For each gene, expression levels were normalized to GAPDH as the control. Relative expression levels were calculated with the 2-ΔΔCt method, comparing the expression level of the gene of interest between the test sample and control sample.

### XTT Cell Proliferation Assay

XTT Cell Proliferation Assay Kit II (Roche) was used according to manufacturer’s instruction. The stable Huh7 cells were seeded into 96-well cell culture plates at a concentration of 4 × 10^3^ cells per well. The cells were incubated at 37 °C incubator for 3 days. At the day of the experiment, the XTT labeling reagent was mixed with an electron-coupling reagent in a 50:1 ratio. 50 μl of XTT labeling mixture was added per well and the cells were incubated with this mixture for 2 h at 37 °C. The spectrophotometric absorbance of the samples at 475 nm were measured in a plate reader. A second measurement was performed at 650 nm as the reference wavelength.

## Results

The candidate transcriptional targets of the canonical Wnt/β-catenin pathway were determined previously in our laboratory by using genome wide microarray analyses and SAGE techniques. MGAT1 gene has been included among the candidate Wnt/β-catenin target genes based on the data obtained from SAGE and Microarray analyses. It is one of the genes that were shown to be upregulated in response to mutant β-catenin overexpression in the Huh7 cell line [15].

### MGAT1 is upregulated in response to Wnt/β-catenin pathway activation

**Fig. 1:**
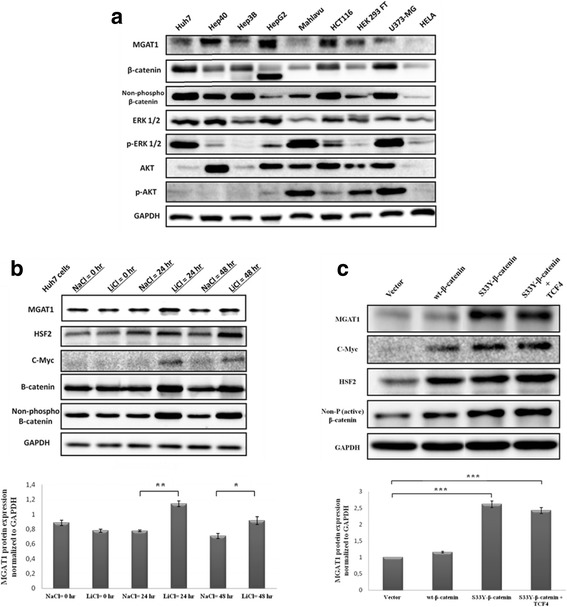
Western Blotting for determination of basal expression levels of the indicated proteins in various carcinoma and non-carcinoma cell lines (**a**). Treatment of Huh7 cells with NaCl (control) and LiCl in time-dependent manner- 0, 24 and 48 h. Densitometry of the bands in the Western Blot shows MGAT1 protein levels normalized to GAPDH (**b**). Overexpression of wild-type and mutant β-catenin in Huh7 cells and western blotting to detect the protein levels of the indicated proteins. Densitometry of the bands shows MGAT1/GAPDH protein levels relative to control (vector-only) transfection (**c**). Each graph is representative of at least two independent experiments. Error bars: SD, (*p value < 0.05; ** *p* value < 0.01; *** *p* value < 0.001)

The basal protein levels of MGAT1, along with a few other key proteins of cell proliferation were determined in various cell types, including carcinoma and non-carcinoma cell lines (Fig. 1a). We observed higher protein expression levels of MGAT1 in hepatocellular carcinoma cells (Hep40, Hep3B and HepG2) and colorectal carcinoma cells (HCT116) compared to other cell lines, such as Mahlavu, HEK 293FT and HeLa. It is noteworthy that hepatocellular carcinoma and colon carcinoma are the two cancer types in which the deregulation of Wnt/β-catenin signaling is mostly observed [10].

Lithium chloride (LiCl) is a widely used GSK-3β inhibitor that results in the activation of Wnt/β-catenin signaling. It interferes with the function of GSK-3β degradation complex and prevents β-catenin phosphorylation and degradation [16]. Huh7 cells were treated with LiCl and NaCl (as control) in a time-dependent manner (0 h, 24 h and 48 h). We showed that 24-h and 48-h of LiCl treatment result in increased total β-catenin and non-phosphorylated (active) β-catenin protein levels (Fig. 1b). c-Myc and Heat Shock Factor 2 (HSF2) are the known targets of β-catenin [15, 17]. In our analysis the treatment of LiCl caused an increase in the levels of c-Myc protein at the 24 h and 48 h treatments with LiCl, whereas HSF2 protein accumulated mostly at the 48 h. Also, the level of MGAT1 protein increased at the 24 h and 48 h treatments with LiCl, which is reminiscent of the impact of activated β-catenin pathway in cells (Fig. 1b).

Unlike other hepatocellular carcinoma cell lines, Huh7 cells do not show abnormal activity of Wnt/β-catenin pathway. In order to activate the pathway, Huh7 cells were transfected with the plasmids expressing either the wild-type β-catenin, S33Y-β-catenin or the combination of S33Y-β-catenin and TCF4. The protein levels of MGAT1 were determined by Western Blotting (Fig. 1c). HSF2 and c-Myc proteins are well established targets of Wnt/β-catenin pathway therefore they were used as positive controls. Non-phosphorylated β-catenin specifically recognizes β-catenin only when Serine 45 residue is not phosphorylated or not targeted for degradation; therefore, it indicates the active β-catenin levels. We also observed the higher accumulation of MGAT1 upon overexpression of S33Y-β-catenin, and the combination of S33Y-β-catenin with TCF4 in transfected cells. Likewise, overexpression of either wild-type β-catenin or S33Y-β-catenin led to an increase in the protein levels of both c-Myc and HSF2. It is worth mentioning that c-Myc displayed almost no protein expression in the cells transfected with the empty vector. Taken together, our results clearly demonstrate that MGAT1 protein is concurrently regulated with the activation of β-catenin (Fig. 1c).

### MGAT1 mRNA levels increase upon Wnt/β-catenin pathway activation

In order to determine MGAT1 mRNA levels in response to Wnt/β-catenin pathway activation, Huh7 cells were treated with either Wnt agonist or GSK3β inhibitor XII (TWS 119). TWS 119 is a synthetic chemical inhibitor of GSK3β and used commonly for the activation of Wnt/β-catenin signaling [18]. Quantitative real-time PCR was performed to detect MGAT1 mRNA levels. AXIN2, being a widely known target of Wnt/β-catenin pathway, was used as positive control.

**Fig. 2:**
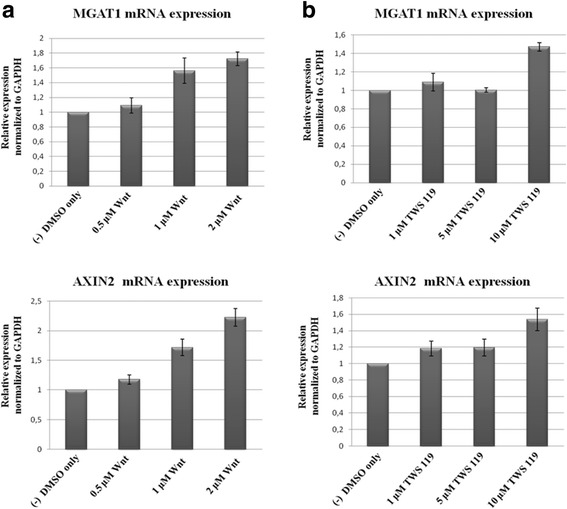
Quantitative real-time PCR analysis for the determination of MGAT1 mRNA levels in Huh7 cells treated with different concentrations of Wnt agonist (**a**) and TWS 119 (**b**). mRNA levels of AXIN2 were also determined as positive control. Relative expression levels of MGAT1 and AXIN2 were obtained by normalization to GAPDH expression levels

The results of q-RTPCR indicate that MGAT1 mRNA levels increased gradually with increasing concentrations of the Wnt agonist. As expected, AXIN2 mRNA was upregulated by Wnt agonist treatment (Fig. 2a). MGAT1 mRNA levels displayed a marked increase only in the cells treated with 10 μM TWS 119, but not in other concentrations. AXIN2 mRNA was also upregulated most in response to the same concentration of TWS 119 (Fig. 2b).

### MGAT1 shows increased promoter activity upon β-catenin overexpression.

To get insights into the transcriptional regulation of MGAT1 gene under modulated Wnt/β-catenin pathway activation we performed luciferase reporter assay to monitor the promoter activity of this gene. For positive control of luciferase activity, TOPFLASH vector which contains three copies of wild-type TCF4 binding sites was used. On the other hand, FOPFLASH vector which contains mutant TCF4 binding sites, was used as negative control. The ratio of firefly luciferase to renilla luciferase was determined for each case, and plotted as relative luciferase activity.

**Fig. 3:**
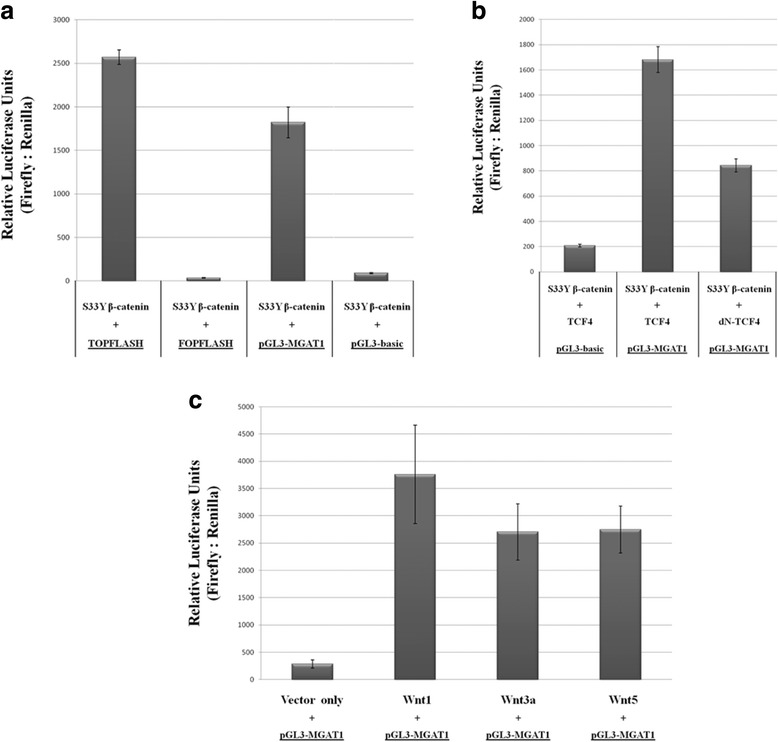
Luciferase reporter assay on Huh7 cells in the presence of S33Y-β-catenin overexpression (**a**), Huh7 cells overexpressing S33Y-β-catenin in combination with either TCF4 or dN-TCF4 (**b**), Huh7 cells overexpressing the three canonical Wnt ligands – Wnt1, Wnt3a and Wnt5 (**c**). Error bars: SE. Each graph is representative of at least two independent experiments

Overexpression of the degradation-resistant β-catenin mutant (S33Y-β-catenin) in Huh7 cells resulted in higher luciferase activity from MGAT1 promoter compared to the negative control vector FOPFLASH, or the promoterless luciferase vector pGL3-basic (Fig. 3a).

Furthermore, S33Y-β-catenin was overexpressed in Huh7 cells in combination with either wild-type transcription factor TCF4 or its dominant negative form (dN-TCF4). Relative luciferase activity from MGAT1 promoter was determined together with the activity from the promoterless pGL3-basic vector as the negative control. Overexpression of S33Y-β-catenin together with TCF4 led to more than 8-fold increase in MGAT1 promoter activity; however, when dominant negative form of TCF4 (dN-TCF4) is overexpressed with S33Y-β-catenin, this activity was reduced nearly in half (Fig. 3b).

In order to activate the pathway, three different ligands of the canonical Wnt/β-catenin signaling pathway (Wnt1, Wnt3a and Wnt5) were overexpressed in Huh7 cell lines by transfecting the cells with each of the pLNCX-Wnt1, pLNCX-Wnt3a and pLNCX-Wnt5 plasmids. Relative luciferase activity from the MGAT1 promoter was determined in each case. Overexpression of Wnt1 led to the highest luciferase activity from MGAT1 promoter (approximately 15-fold increase compared to control), whereas overexpression of Wnt3a and Wnt5 also led to more than 10-fold increase in luciferase activity compared to the one obtained from mock transfection (Fig. 3c). As a result of these series of experiments, MGAT1 promoter activity was found to be higher in response to increased Wnt/β-catenin activity in all the different approaches used.

### Assessing the migration and proliferation abilities of MGAT1-expressing stable cell lines

Stable expression of MGAT1 and GFP (as negative control) genes in Huh7 cells was established as a first step, in order to analyze the effect of MGAT1 both in vitro and in vivo.

**Fig. 4:**
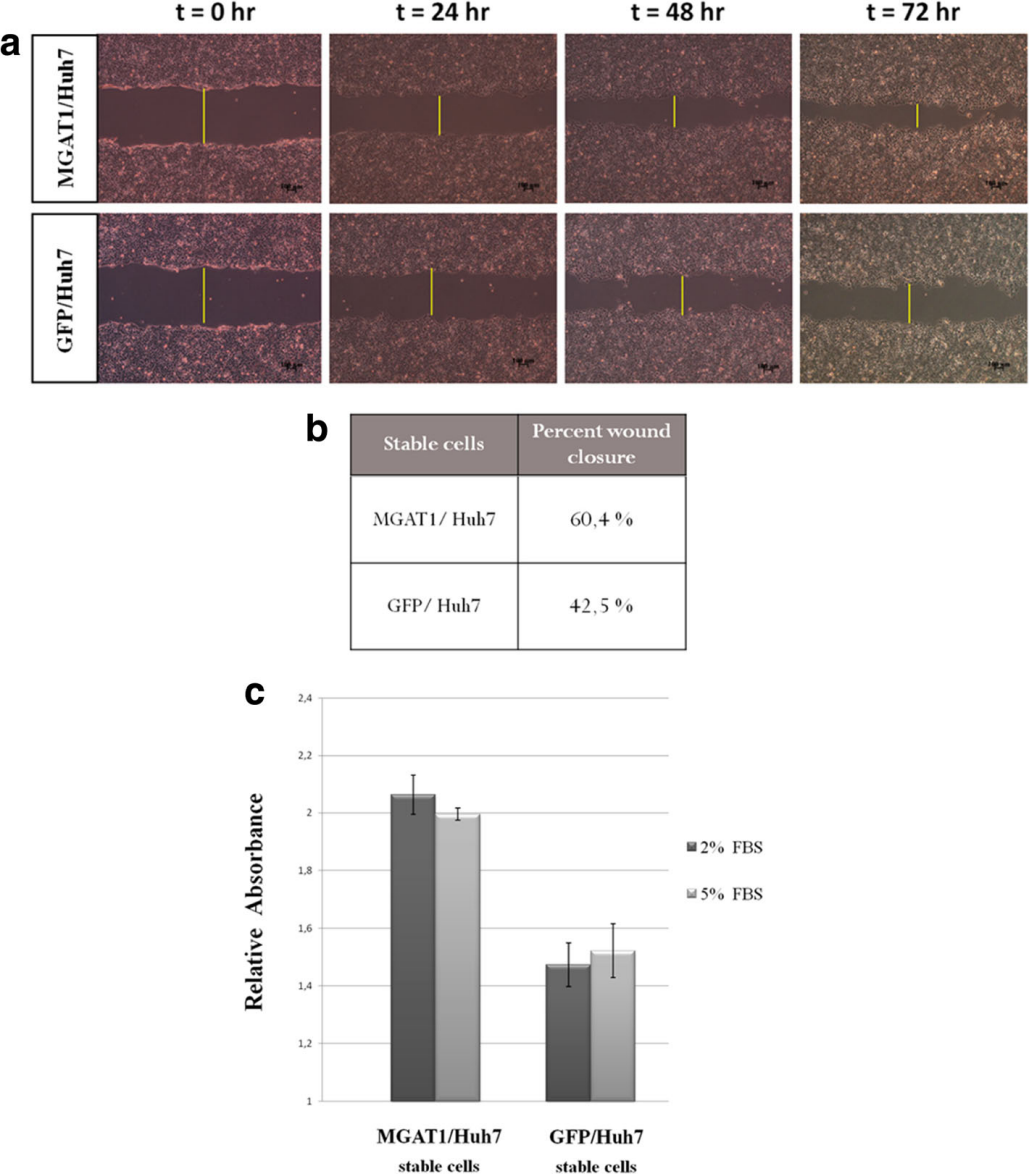
Wound healing assay for the stable cells overexpressing the MGAT1 and GFP (control) genes. The bright field images are representative of two independent experiments (**a**). The distances across the wound are measured by Image J and the differences are presented as percent wound closure (**b**). MGAT1 stable expression in Huh7 cells results in higher proliferation rate compared to GFP-expressing control stable cells, as determined by XTT assay. The graph indicates results from two independent experiments (**c**)

Wound healing assay was performed in order to determine whether there existed any difference in the cell migration capacities of the stable Huh7 cells. MGAT1 and GFP overexpressing Huh7 cells were used for this assay. After introducing a scratch across the confluent and adherent layer of cells in 6-well plates, the cells were visualized under the microscope at different time points starting from 0 h up to 72 h. The widths of the scratches indicated by yellow lines were quantified by Image J software (Fig. 4a).

Differences between wound closure capacities of the stable cells were observed after 72 h. Distances across the wound were measured at each time point, and difference between the initial and final values were computed as percent wound closure for each stable cell line. Overall, MGAT1 overexpressing stable Huh7 cells demonstrated a higher percentage of wound closure (60,4%) compared to the cells overexpressing GFP as the control (42,5%) (Fig. 4b). These results substantiate the fact that MGAT1 overexpressing cells have higher proliferative capacity compared to control cells.

In order to determine whether there is any difference in the cellular proliferation properties of the stable cell lines, we perform XTT Assay. The stable Huh7 cell lines overexpressing MGAT1 and GFP (control) were grown at 96-well culture plates for three days under two different sets of serum starvation conditions (2% FBS and 5% FBS). At the end of three days, absorbance spectra for the cells treated with XTT reagent was recorded at 475 nm using an ELISA plate reader. Normalization was done by taking another set of measurements at the reference wavelength of 650 nm. The results indicate that the relative absorbance values are approximately 30% higher in MGAT1 overexpressing cells compared to the GFP overexpressing cells in both of the serum starvation conditions (Fig. 4c).

### In vivo analysis of tumorigenesis

**Fig. 5:**
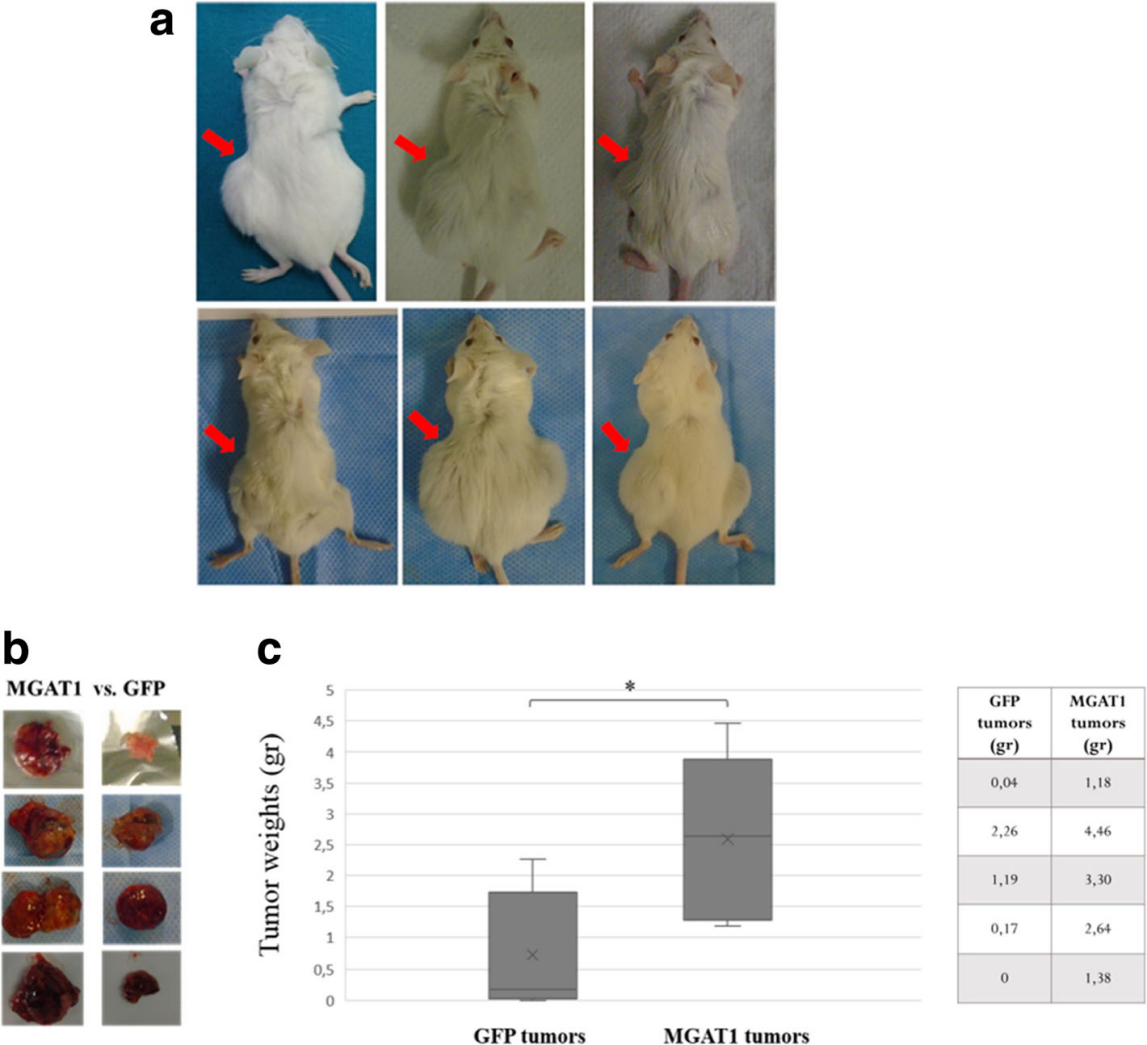
MGAT1 overexpressing stable cells are subcutaneously injected into the left flank region of each mouse (indicated by the red arrows). GFP overexpressing stable cells are injected into the right flank as control (**a**). Representative tumor images were obtained for comparison (**b**). Box plots of tumor weights of two mice groups corresponding to GFP and MGAT1 tumors and the weights are depicted as table in the right panel (**c**). Error bars: SD, (*p value < 0.05). The graph indicates results from 5 SCID mice for each group

In order to test the tumor forming abilities of the stable Huh7 cell lines in vivo, we performed Xenograft assays in SCID mice. The mice were injected subcutaneously and bilaterally from their flank regions. MGAT1 overexpressing Huh7 cells were injected on the left flank of the mice, while GFP overexpressing Huh7 cells were injected on the right flank as the control (Fig.5a).

The mice were checked for tumor formation regularly. These experiments clearly indicate visible differences in tumor sizes 3–4 weeks after the injection of MGAT1 overexpressing Huh7 cells into the mice. At this point, the mice were sacrificed and tumors were isolated (Fig. 5b). Differences between the tumors were determined by weighting, and the average weights for each set are plotted (Fig. 5c). The tumors formed by MGAT1 overexpressing cells were found to be more than 3 times larger than the control tumors.

## Discussion

The canonical Wnt/β-catenin signaling pathway is a highly conserved pathway and it is involved in various differentiation events during embryonic development, such as axis formation, cellular proliferation, differentiation and morphogenesis. β-catenin is considered to be the key molecule in this pathway. In addition to its functions in vertebrate early development, Wnt/β-catenin pathway has the potential to initiate tumor formation when aberrantly activated. Those characteristics of the Wnt/β-catenin signaling pathway makes the pathway itself and its targets important subjects in cancer research fields. In fact, the genes regulated by this pathway are potential drug and gene therapy targets. Therefore, we previously performed SAGE and microarray screens in our laboratory in order to identify novel transcriptional targets of the Wnt/β-catenin pathway. As the result of these analyses, a number of genes were determined to be either upregulated or downregulated significantly in an experimental setup mimicking the constitutively active Wnt/β-catenin pathway.

MGAT1 was one of the genes that showed differential expression levels in response to β-catenin activation. Moreover, it was previously included among the candidate target genes of Wnt/β-catenin signaling in SAGE analyses [15]. MGAT1 is known to code for an enzyme essential for the synthesis of hybrid and complex N-glycans, but was not associated with carcinogenesis until now.

To address this more closely, we used various carcinoma cell lines (mostly hepatocellular carcinoma) and non-carcinoma cell lines to monitor the basal protein expression levels of MGAT1 alongside other important key cell proliferation proteins (Fig. 1a). MGAT1 protein was detected mostly in hepatocellular carcinoma and colon carcinoma cell lines. We therefore hypothesized that MGAT1 can be a novel transcriptional target of the Wnt/β-catenin pathway as hepatocellular carcinoma and colon carcinoma are the two types of carcinoma which display the highest Wnt/β-catenin pathway activation [10]. This can also be seen from the protein expression levels of non-phospho (active) β-catenin, which is higher in hepatocellular carcionoma and colon carcinoma cell lines. Treatment of Huh7 cells with LiCl indicated the upregulation of MGAT1 protein at 24 and 48 h, which correlated with the increase in active β-catenin levels. Overexpression of either S33Y-β-catenin by itself or in combination with TCF4 also caused an increase in the protein levels of MGAT1. Furthermore, the promoter activity of MGAT1 was found to be increased in response to Wnt/β-catenin pathway activation either by overexpressing the stable mutant form of β-catenin, or the canonical Wnt ligands in Huh7 cells.

To better complement these findings, a series of cell migration and proliferation assays were performed to assess the proliferative potentials of the stable cell lines in vitro. The results of wound healing assay suggest that MGAT1 overexpressing stable cells were more effective in closing the wound compared to GFP overexpressing cells, which gives us a hint about their higher migration and proliferation abilities. The stable cell lines were also analyzed by XTT cell proliferation assay to test for proliferation related to metabolic activities of the cells. At normal growth conditions (10% FBS) there appeared to be no significant difference between the absorbance values of the stable cell lines (data not shown). However, under serum starvation conditions (2% or 5% FBS), MGAT1 overexpressing cells displayed higher proliferation rate compared to GFP overexpressing cells.

To further substantiate the impact of MGAT1 on tumorigenesis, and assess its role in vivo tumorigenesis, the stable cell lines were subsequently used for xenograft assays. The injections were made subcutaneously and bilaterally such that the left flank regions of mice were injected with MGAT1 overexpressing cells, whereas the right flank regions were injected with control cells (GFP overexpressing Huh7 cells). In vivo xenograft experiments demonstrated that MGAT1 overexpressing cells were able to generate larger tumors compared to GFP overexpressing cells.

MGAT1 is one of the proteins functioning in the process of N-glycan synthesis, which play important roles in cell-cell and cell-matrix interactions essential for the development of multicellular organisms. Proteoglycans are the key regulators of cell-matrix dynamics and there are numerous studies showing that abnormal or erroneous expression of proteoglycans can lead to various types of carcinoma [19–22]. Within this context, it is reasonable to suggest that MGAT1 overexpression can interfere with the regular proteoglycan synthesis mechanisms, and therefore, might have the potential to enhance tumor progression.

## Conclusions

Taken together, we identified for the first time that MGAT1 gene is a novel transcriptional target of Wnt/β-catenin pathway. Furthermore, we have shown MGAT1 to be upregulated in response to Wnt/β-catenin pathway activation. Since this pathway is one of the most important intracellular pathways for cancer progression, we tested this novel target gene in terms of its tumorigenic capacity. As a result, we were able to characterize MGAT1 gene as a potential oncogene, since the overexpression of this gene in hepatocellular carcinoma cell lines leads to the formation of larger tumors when transplanted into SCID mice. Future studies would need to be carried out, such as transcriptomic analysis of the tumors by RNA Sequencing technique, in order to determine which pathways and biological processes contribute to these mechanisms.

## Abbreviations

MGAT1: Mannosyl (alpha-1,3-)-glycoprotein beta-1,2-N-acetylglucosaminyl-transferase
QRTPCR: Quantitative Reverse Transcription Polymerase Chain Reaction
SAGE: Serial Analysis of Gene Expression
SCID: Severe Combined Immunodeficiency
